# A Custom Keyword Tool for Improving the Quality of Social Media Monitoring on Vaccine Safety: A Proof of Concept

**DOI:** 10.3389/ijph.2025.1608480

**Published:** 2025-08-21

**Authors:** Lucie Marisa Bucci, Smaragda Lamprianou, Francesco Gesualdo, Shanthi Pal

**Affiliations:** ^1^ Bucci-Hepworth Health Services, Pincourt, QC, Canada; ^2^ World Health Organization, Geneva, Switzerland; ^3^ Department of Translational Research and New Technologies in Medicine and Surgery, University of Pisa, Pisa, Italy

**Keywords:** misinformation related to health, social media monitoring, vaccine safety, vaccine acceptance, vaccine hesitancy

## Background

Social media monitoring is one of several ways for health authorities to capture specific insights into population perceptions about vaccine safety [1]. Commercial and open-source tools are helpful for gathering data on socio-cultural, religious, and political trends, but also for detecting what is being said about vaccine safety and why populations are delaying or refusing vaccination [2]. Monitoring and tracking digital forums for target audiences and influencers and identifying misinformation provides additional understanding. However, despite the clear need, public health authorities globally are severely constrained in their capacity to effectively address the overwhelming volume and complexity of misinformation [3]. To complicate matters, international, national, and corporate infodemic management policies are imposing information users to change the way they share information online and on social media platforms. Recent examples of how information users are adapting include the use of memes and increasing the amount and speed of information disseminated between platforms. This rapid evolution of misinformation tactics and limited public health resources including insufficient staffing, and a lack of specialized digital capacity within many public health authorities, renders comprehensive oversight incredibly challenging. As digital information environments become more complex, existing tools for social media monitoring need to adapt to meet the needs of public health authorities that may not have the resources to undertake comprehensive social media listening [4]. For health authorities to truly benefit from better quality social media intelligence, innovations must be developed that are not only required but also accessible, adaptable and feasible to implement [5].

The Vaccine Safety Net (VSN), the World Health Organization’s (WHO) global network that facilitates access to trustworthy, science-based vaccine safety information, identified this challenge as an opportunity to contribute to a rapidly expanding area. The VSN consists of member websites seeking to achieve more effective ways for communicating through digital and social media analytics research [6]. The latter involves identifying high impact vaccine safety related issues on social media for predicting and pre-bunking misinformation, developing and testing social media messages, as well as assessing their relevance and impact using a commercial platform with a social listening tool. Leveraging the VSN’s expertise in social medial listening, we developed and tested a custom keyword filter designed for adaptable global implementation by public health authorities. This filter aims to address the significant challenge posed by the sheer volume and evolving nature of misinformation, which often overwhelm existing commercial and online generic filters and their ability to provide precise results. For example, many generic keyword filters rely on estimations and are capable of over-filtering (or under-filtering) valuable social media content and fail to capture relevant information. Social media content is highly contextual and generic keyword filters do not interpret the nuances of language (e.g., comedy, sarcasm, anger, etc.)

## Objective

We sought to understand how to optimize social media searches on vaccine safety using a custom keyword filter for better quality search results. A proof-of-concept project whereby a custom keyword filter was designed using Kim et al.’s [7] conceptual framework and tested using a commercial social listening platform and open source artificial intelligence (AI) tools with the intent of analyzing the quantity of irrelevant relevant mentions retrieved from vaccine safety searches on X^®^ in Canada, United States, Italy and United Kingdom.

Unfiltered keywords can yield large amount of irrelevant data [8]. Therefore, custom keyword filters are meaningful methods for improving digital and social media monitoring practices in response to constantly evolving information environments. For additional accuracy, we chose to first create and test a keyword filter with vaccine -related keywords. A vaccine safety keyword filter was subsequently created and tested to distill the information. We added artificial intelligence (AI) derived keywords to the filter, which expanded the social media datasets.

## Methods

The custom keyword filter involves three steps: 1) frequency screening; 2) sampling; and 3) search implementation.

### Frequency Screening

A list of candidate vaccine and vaccine safety keywords were pooled in collaboration with VSN members from Canada, Italy, United States and United Kingdom. Candidate keywords were selected considering native language of targeted countries, media reports, published literature and epidemiological events. The list of candidate keywords was applied to a six [6] month retrospective scan of X^®^ conversations between January and June 2023 using a commercial social media monitoring platform. We tracked keywords that peaked on X^®^ and developed a history of trending candidate keywords for this period. We used this dataset to identify the frequency of candidate keywords. Candidate keywords that had less than 30% of mentions per month were discarded from the keyword list. This frequency threshold was selected through team consensus for this proof of concept. This initial triage was used to identify vaccine and vaccine safety keywords used more regularly in X^®^ conversations.

A data analyst was enlisted to assist with AI keyword identification. Additional vaccine and vaccine safety-related keywords were extracted from the original dataset using Bidirectional Encoder Representations from Transformers (BERT) and Generative Pre-trained Transformer 3 (GPT-3).

### Sampling

The remaining candidate keywords were then screened for relevance. To do this, a real time dataset of X^®^ keyword mentions of up to one [1] week was generated. We sampled two hundred (200) mentions for each candidate keyword, including reposts using a commercial sample operator. Three team members, experts in vaccination and vaccine safety, reviewed each sample to determine relevance. We assessed the relevance of keywords by classifying their sensitivity and specificity to “vaccine-related” and then “vaccine safety-related” words. Keywords were considered relevant if they were used in the context of vaccination conversations or was implicit in meaning.

Keywords that returned no relevance were removed from the list but added to a “negative keyword list” for prospective Boolean searches. Duplicate posts within each keyword dataset were also removed. Other content in posts such as emojis, hashtags and usernames were not considered relevant. Links to websites, if included in posts, were used to clarify context of post. Replies were also excluded. Lastly, common words known as “stop-words” (e.g., the, she, he, it, are, etc.) were excluded.

### Search Implementation

Keywords determined to be highly relevant were not used in a new prospective search on X^®^ to evaluate the quality of their search results. This final step was not part of the scope of this proof of concept.

## Results

**FIGURE 1:**
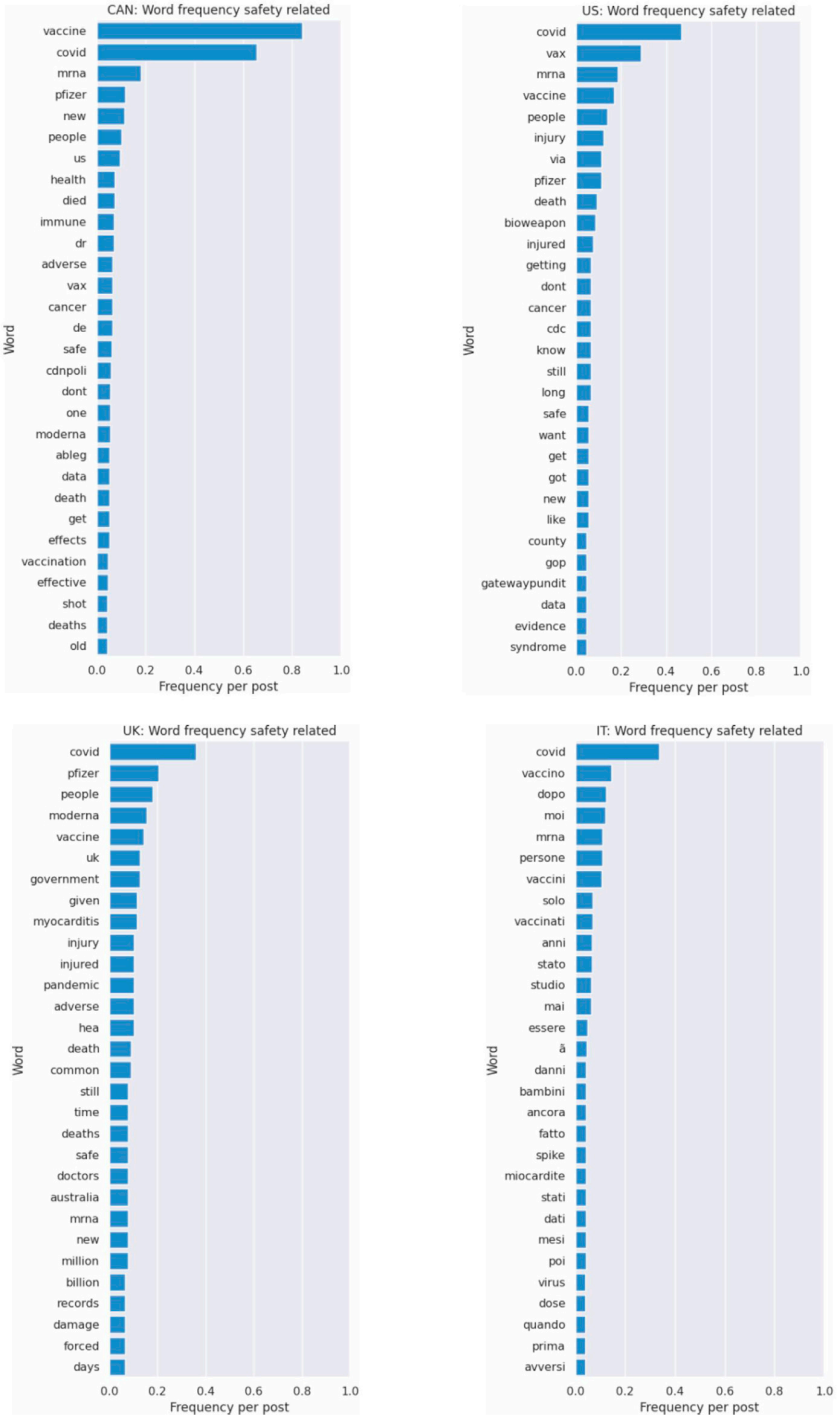
Keyword visualization: Vaccine safety related posts (Canada & United States (top), United Kingdom & Italy (bottom)) (Switzerland, 2024).

**FIGURE 2:**
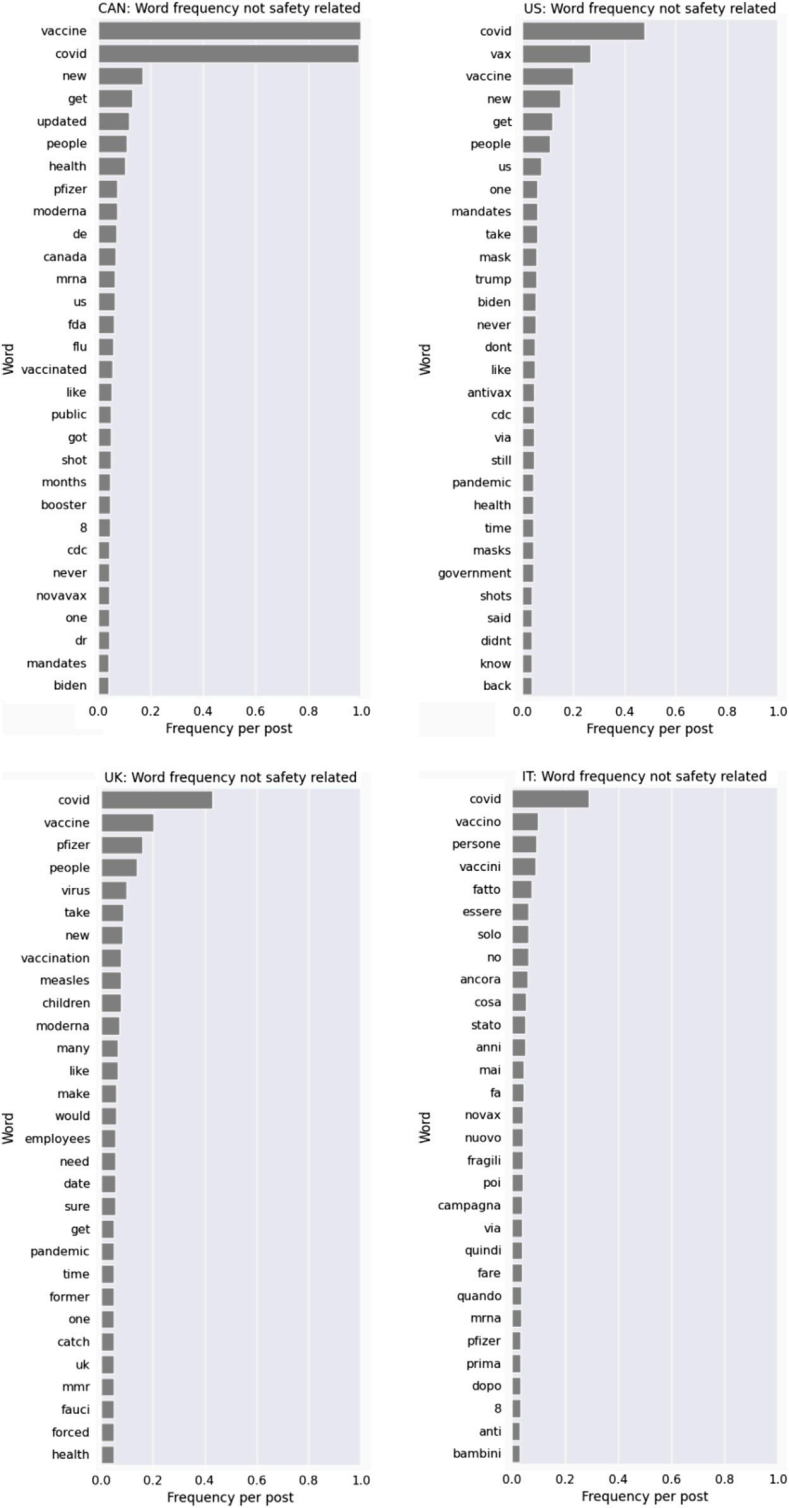
Keyword visualization: Vaccine related but not safety related posts (Canada & United States (top), United Kingdom & Italy (bottom)) (Switzerland, 2024).

Thirty (30) vaccine and vaccine safety-related keywords were extracted for each country (see Figures 1, 2) using manual and AI methods. Vaccine-related keywords were used in three thousand one hundred and seventy-eight (3178) posts. While vaccine safety-related keywords were used in eight-hundred and sixty-nine (869) posts (see Table 1). Bert and GPT identified additional keywords including combined terms not previously identified. Themes extracted and analysed from vaccine safety-related mentions include public skepticism about vaccine safety, particularly COVID-19 vaccines, polarization between vaccination perspectives, concerns about misinformation, mistrust in government, influencers, and pharmaceutical companies.

**TABLE 1:** Number of posts per country (Switzerland, 2024).

Country	Filter	# Vaccine related	# Vaccine safety related
CAN	COVID-19 vaccine	199	37
CAN	Coronavirus	53	13
CAN	COVID vaccines	193	41
CAN	Side effects	92	76
CAN	AstraZeneca	101	58
CAN	Pfizer	142	63
CAN	Pandemic	4	0
CAN	Plandemic	32	4
CAN	Total	816	292
USA	COVID	13	2
USA	COVID-19	45	4
USA	Coronavirus	46	6
USA	Vaccine	188	42
USA	Vax	182	28
USA	Vaxx	182	25
USA	Total	656	107
UK	Vaccine	117	45
UK	Vaccine	39	2
UK	COVID	29	14
UK	Jabs	60	12
UK	Coronavirus	18	5
UK	Total	263	78
IT	Vaccino	200	72
IT	Vaccini	194	76
IT	Vaccinato	184	30
IT	Vaccinata	114	24
IT	Vaccinati	190	57
IT	Vaccinate	139	36
IT	Vaccinare	196	17
IT	Vaccinazion	200	69
IT	Immunizzazione	44	10
IT	Pfizer	91	36
IT	COVID19	25	9
IT	COVID-19	66	28
IT	Total	1443	392

### Conclusion

Our objective was to develop a custom keyword filter for producing better quality social media intelligence for public health authorities to easily use and be versatile in their vaccine safety communication strategies. We found the development of a custom keyword filter that uses both manual and AI as methods for extracting social media mentions and performing content analysis about vaccine and vaccine safety-related posts yielded quality data. The strategy for our proof-of-concept study used a commercial platform for testing and keyword refinement. We anticipate that custom keyword filters may be used with other commercial and freely available keyword filters for more precise results. This is an advantage over using commercial or online filters alone. That said, keyword filter refinement is a time-consuming process as well as data analysis and interpretation. Despite these shortcomings, social media monitoring innovations are needed to keep with changing information environments. While we did not test the keyword filter for sensitivity, we found the filter to meet our expectations for specificity. New tools need to focus on improving the relevance of outputs. AI offers other avenues for filtering candidate keywords but we are still learning about its limitations. Our proof-of-concept project contributes to a rapidly evolving area and provides new insights on how public health can use adaptable keyword filter tools, in addition to commercial tools, to improve their capacity to respond to online misinformation.
